# Diversity and population structure of *Plasmodium falciparum* in Thailand based on the spatial and temporal haplotype patterns of the C-terminal 19-kDa domain of merozoite surface protein-1

**DOI:** 10.1186/1475-2875-13-54

**Published:** 2014-02-12

**Authors:** Phumin Simpalipan, Sittiporn Pattaradilokrat, Napaporn Siripoon, Aree Seugorn, Morakot Kaewthamasorn, Robert DJ Butcher, Pongchai Harnyuttanakorn

**Affiliations:** 1 Department of Biology, Faculty of Science, Chulalongkorn University, Bangkok 10330, Thailand; 2 College of Public Health Sciences, Chulalongkorn University, Bangkok 10330, Thailand; 3 Department of Pathology, Faculty of Veterinary Science, Chulalongkorn University, Bangkok 10330, Thailand; 4 Office of Research Affairs, Faculty of Science, Chulalongkorn University, Bangkok 10330, Thailand

**Keywords:** Allelic polymorphism, DNA sequencing, Merozoite surface antigen, Population structure, Southeast Asia

## Abstract

**Background:**

The 19-kDa C-terminal region of the merozoite surface protein-1 of the human malaria parasite *Plasmodium falciparum (Pf*MSP-1_19_) constitutes the major component on the surface of merozoites and is considered as one of the leading candidates for asexual blood stage vaccines. Because the protein exhibits a level of sequence variation that may compromise the effectiveness of a vaccine, the global sequence diversity of *Pf*MSP-1_19_ has been subjected to extensive research, especially in malaria endemic areas. In Thailand, *Pf*MSP-1_19_ sequences have been derived from a single parasite population in Tak province, located along the Thailand-Myanmar border, since 1995. However, the extent of sequence variation and the spatiotemporal patterns of the MSP-1_19_ haplotypes along the Thai borders with Laos and Cambodia are unknown.

**Methods:**

Sixty-three isolates of *P. falciparum* from five geographically isolated populations along the Thai borders with Myanmar, Laos and Cambodia in three transmission seasons between 2002 and 2008 were collected and culture-adapted. The *msp*-1 gene block 17 was sequenced and analysed for the allelic diversity, frequency and distribution patterns of *Pf*MSP-1_19_ haplotypes in individual populations. The *Pf*MSP-1_19_ haplotype patterns were then compared between parasite populations to infer the population structure and genetic differentiation of the malaria parasite.

**Results:**

Five conserved polymorphic positions, which accounted for five distinct haplotypes, of *Pf*MSP-1_19_ were identified. Differences in the prevalence of *Pf*MSP-1_19_ haplotypes were detected in different geographical regions, with the highest levels of genetic diversity being found in the Kanchanaburi and Ranong provinces along the Thailand-Myanmar border and Trat province located at the Thailand-Cambodia border. Despite this variability, the distribution patterns of individual *Pf*MSP-1_19_ haplotypes seemed to be very similar across the country and over the three malarial transmission seasons, suggesting that gene flow may operate between parasite populations circulating in Thailand and the three neighboring countries.

**Conclusion:**

The major MSP-1_19_ haplotypes of *P. falciparum* populations in all endemic populations during three transmission seasons in Thailand were identified, providing basic information on the common haplotypes of MSP-1_19_ that is of use for malaria vaccine development and inferring the population structure of *P. falciparum* populations in Thailand.

## Background

Because the clinical symptoms and pathology of malarial infections are caused by the erythrocytic stage cycles of the *Plasmodium* parasites [1-3], the development of a malaria vaccine targeting the asexual blood stage has a high priority in current malaria research. Safe and effective malaria vaccines are greatly needed in malaria endemic areas and could be used to supplement strategies of vector control and medical treatment for reducing the rates of morbidity and mortality [4]. The estimated number of malaria cases in 2012 was over 200 million with about 660,000 deaths [5], mostly in children under five years of age and pregnant women with heavy exposure to malaria infections. These numbers are, however, expected to increase due to treatment failure and/or delay in parasite clearance associated with the evolution and spread of drug-resistant malaria parasites [6]. To date, a small number of vaccines against the blood stage malaria parasites have been developed and a few of these are currently undergoing large-scale clinical trials [7,8]. Despite the fact that the majority of the vaccines have been shown to be safe and highly effective in animal models, immunizations with these blood stage malaria vaccines in humans was found to elicit allelic-specific anti-parasitic immunity [9-11], thereby compromising the efficacy of these vaccines. This is due to the allelic diversity of the strongly antigenic portions used as candidate vaccine antigens in the natural parasite populations, which poses one of the major challenges to development of effective vaccines. Thus, it is essential to investigate the extent of the genetic variation of malarial antigens and the population structure of malaria parasites in nature so that a better malaria vaccine, such as those that incorporate multiple variants of protective antigens can be properly designed. Alternatively, the development of malaria vaccines based on the conserved epitopes of antigens, if they are sufficiently antigenic and so elicit protective immunity, would offer an alternative strategy.

Components of blood stage malaria vaccines are generally derived from parasite ligands (antigens) expressed on the surface of merozoites, the only extracellular blood stage parasite. Among them is the merozoite surface protein-1 (MSP-1) that is currently used as a major component of anti-blood stage human malaria vaccines [12,13]. The gene encoding MSP-1 of the human malaria parasite *Plasmodium falciparum* spans ~5 kb in size on chromosome 9 and can be divided into 17 blocks, according to the presence or absence of 9 bp repeats and the type of repeating sequences [14,15]. The nucleotide sequences of the *msp*-1 gene are classified into two allelic types: K1 and MAD20, with the exception of the highly polymorphic block 2 that is represented by at least three allelic types (K1, MAD20 and RO33) [16]. The MSP-1 protein is initially synthesized as a 190 kDa or 195 kDa polypeptide [17]. During the beginning of merozoite invasion into the host’s erythrocyte, the protein undergoes primary proteolytic cleavage events to generate four fragments of 83 kDa (MSP-1_83_), 30 kDa (MSP-1_30_), 38 kDa (MSP-1_38_) and 42 kDa (MSP-1_42_) [18,19]. These fragments are held together as a complex and attached to the parasite’s surface via a glycosylphosphatidylinositol anchor. At the point of merozoite invasion, the MSP-1_42_ fragment is proteolytically processed to form the 33 kDa (MSP-1_33_) and 19 kDa (MSP-1_19_) fragments [20]. The MSP-1_19_ fragment, containing two epidermal growth factor (EGF)-like domains, remains attached to the parasite’s surface [21].

Two coding regions of the *msp*-1 gene (blocks 2 and 17) have been identified to be prime targets of protective immunity. Previous studies have indicated that natural clinical immunity against blood malaria infections targets the N-terminal fragments encoded by block 2 of *msp*-1 gene in an allelic-type specific manner [22-24]. Naturally acquired antibodies to the C-terminal MSP-1_19,_ encoded by block 17 of the *msp*-1 gene, could also inhibit erythrocyte invasion by preventing the secondary processing that released MSP-1_19_ from the rest of the MSP-1 complex [20,25]. Invasion-inhibiting antibodies against MSP-1_19_ have been detected in the sera from individuals living in areas that the highly endemic for malaria and were associated with protection from clinical malaria [26-33]. As mentioned, a few MSP-1_19_-based vaccines are currently in Phase I and Phase II clinical trials in humans and so far have been shown to be safe and immunogenic [34-36].

Polymorphism in the *P. falciparum msp*-*1* gene block 17 sequences has been fairly extensively researched and they have been classified into at least 10 MSP-1_**19**_ haplotypes in natural populations of *P. falciparum*[37-51]. Of these, four major haplotypes (Q/KNG/L, E/KNG/L, Q/KNG/L and E/TSR/L) have been frequently identified in multiple malaria endemic regions. Comparisons of the distribution patterns and frequencies of MSP-1_**19**_ haplotypes among different parasite populations revealed that the populations of *P. falciparum* are genetically diverse [46], consistent with the observations based upon the large-scale genome-wide sequencing-based and microarray-based genotyping data [52,53]. In addition, *Pf*MSP-1_19_ haplotypes can also be utilized to infer the population structure of *P. falciparum* parasites. A recent study indicated the subdivision of two *P. falciparum* populations in China according to their variations in the distribution patterns of MSP-1_19_ haplotypes, where the parasite populations in Yunnan (South China) appeared to be more closely related to the parasite populations in Southeast Asia (Thailand and Vietnam) than those in Hainan Island located on the South China Sea [46]. The shared distribution pattern of the haplotypes is consistent with the fact that many reported cases in Yunnan were imported malaria from border areas, suggesting an impediment in the gene flow between the regions in mainland Indochina [54].

Recently, the emergence of artemisinin resistance in *P. falciparum* has been reported in Southeast Asia and has drawn the global attention to the need for improvement in malaria intervention [55,56]. After the first report incidence of artemisinin resistance in *P. falciparum* at the Thailand-Cambodian border in 2008 [57], artemisinin resistance has now been reported on the western border of Thailand close to Myanmar [58]. Because of this spread of artemisinin resistance, it is of great interest to investigate the genetic composition and population structure of Thai *P. falciparum* isolates at these localities. In Thailand, much of the current knowledge of the *msp*-*1* sequence diversity originated from the analyses of a single parasite population at Mea Sod, Tak province at the Thai-Myanmar border [38,39,51], but little work has been done to further elucidate the level of genetic diversity in other regions of the country, especially where the artemisinin resistant parasites are likely to spread. To address this issue, a cross-sectional survey of the allelic diversity of the *msp-1* gene block 17 in five geographically isolated populations of *P. falciparum* in Thailand was performed in order to infer the genetic structure of the parasite populations from the distribution patterns of their MSP-1_19_ haplotypes. The outcome of this work will shed light on the nature and genetic relationship within and between malaria populations and offer a chance to better understand the parasite’s evolution, which will be important for establishing an effective strategy for malaria control and intervention.

## Methods

### Studied areas and preparation of parasites

Field isolates of *P. falciparum* were collected from five localities (1-5), each in a different province, close to the borders of Thailand and three neighbouring countries. These were (1) Ubon Ratchathani, located at the Thailand-Laos border, (2) Trat, located at the Thailand-Cambodia border, and (3–5) Ranong, Kanchanaburi and Mae Hong Son, located at the Thailand-Myanmar border. The subjects were confirmed for *P. falciparum* mono-infection by microscopic examination of Giemsa-stained blood smears. The subjects were recruited in three transmission seasons between: (1) Jun 2002–Oct 2003, (2) Jun 2004–Dec 2005 but also including one subject in 2006 and (3) Jan 2008–Dec 2008 (see Additional file 1). Sixty-three blood stage malaria samples were adapted to i*n vitro* culture and maintained at the Malaria Research Unit, Department of Biology, Faculty of Science, Chulalongkorn University, Thailand, as described previously [59]. Of these, 44 samples collected between 2002 and 2006 were previously genotyped by 12 microsatellite loci and confirmed to be independent clones [59] (see Additional file 1). In brief, the blood stage parasites were cultured in RPMI 1640 medium containing 25 mM HEPES, 4% (v/v) human blood group O erythrocytes (4% haematocrit), 10% (v/v) pooled heat-inactivated serum from healthy donors, 24 mM sodium bicarbonate and 10 μg/mL gentamycin at 37°C with 5% (v/v) CO_2_, 5% (v/v) O_2_ and 90% (v/v) N_2_. The medium was changed daily, and the parasites were allowed to grow to a parasitaemia level of 5-10% and then harvested prior to genomic DNA preparation.

### Preparation of *P. falciparum* genomic DNA

Procedures for preparation of the blood stage malaria parasites and genomic DNA were performed as previously described [60,61]. Briefly, *P. falciparum* infected blood samples were centrifuged at 5,000 *g* and the supernatant was discarded. Then, a 200 μL volume of the packed blood cells was mixed with 500 μL of 0.05% (w/v) saponin solution in phosphate buffered saline (PBS, pH 7.4) to release the blood stage malaria parasites from the human erythrocytes. The parasite pellets were collected by centrifugation at 10,000 *g* for 10 min and washed twice in PBS. The parasites were lysed in a lysis solution (40 mM Tris-HCl, 80 mM EDTA, 2% (w/v) sodium dodecyl sulfate, pH 8.0) containing 2 mg/mL proteinase K. The suspension was sequentially treated with an equal volume of phenol (pH 8.0), phenol/chloroform/isoamyl alcohol (25:24:1 (v/v/v), pH 8.0) and chloroform, harvesting the aqueous phase each time. The genomic DNA was then recovered by ethanol precipitation in the presence of 0.3 M sodium acetate and later dissolved in standard TE buffer (10 mM Tris-HCl, 1 mM EDTA, pH 8.0) and stored at -20°C prior to PCR amplification.

### Amplification and DNA sequencing of the *P. falciparum msp-1* gene block 17

A DNA fragment corresponding to the *msp*-*1* gene block 17 was amplified by the polymerase chain reaction (PCR) with the MM1/17F (5′-TCACAACACCAATGCGTAAAA-3′) and MM1/17R (5′-GAGTATTAATAAGAATGATATTCCTAAG-3′) primer pair, which correspond to nucleotide positions 4,825–4,845 and 5,136–5,109 of the coding sequence of the *msp-1* gene of the *P. falciparum* strain 3D7 (NCBI accession number: XM_001352134, [62]). The PCR amplification reactions were performed in a 50 μL volume containing 200–300 ng of DNA template, 2 mM of MgCl_2_, 200 μM of each dNTP, 0.5 μM each of the forward and reverse primers and 2 units (U) of iTaq™-DNA polymerase enzyme in 1X iTaq PCR buffer (iNtRON Biotechnology, Republic of Korea). Thermal cycling was performed with an optimized profile of an initial denaturation at 95°C for 5 min, followed by 30 cycles of 95°C for 40 s, 53°C for 40 s and 72°C for 40 s, and subsequently a final extension at 72°C for 5 min. PCR products were analysed by standard gel electrophoresis, stained with ethidium bromide and visualized by UV transillumination. All 63 samples generated single amplicons with an expected size of 220 bp, and were subjected to direct Sanger DNA sequencing in both the forward and reverse directions to ensure the accuracy of obtained sequences. Sequencing reactions were performed using the BigDye Terminator v1.1 kit (Applied Biosystems, USA) with an ABI3730 DNA analyser. DNA sequences were manually edited using Bioedit 7.0.0 software and aligned using the MUSCLE Sequence Alignment algorithm in the MEGA 5.2 program [63]. In total, 61 DNA samples generated unambiguous sequences in both directions and were used in the subsequent population genetic analysis.

### DNA sequence analysis and statistical analysis

The predicted amino acid sequences of the C-terminal fragment of *Pf*MSP-1_19_ from the PCR sequenced genomic fragments were aligned with the corresponding sequence of the *P. falciparum* genome reference strain 3D7 using the MUSCLE Sequence Alignment algorithms as above. The extent of genetic diversity was estimated by the number and ratio of MSP-1_**19**_ haplotypes prevalent in the populations. Divergences in the distribution pattern of *Pf*MSP-1_19_ haplotypes between two parasite populations was tested for using the Wright’s fixation index (F_st_) with Arlequin suite version 3.5 [64]. In addition, the departure from the predictions of the neutral mode of molecular evolution was evaluated using three neutrality tests (Tajima’s D, Fu & Li’s D^*^and Fu & Li’s F^*^indices) implement in the DnaSP 5.1 program [65]. Results were deemed to be statistically significant if the *p* value was less than 0.05 (*p* < 0.05).

## Results

### Sequence diversity of *msp-1* gene block 17

**Figure 1:**
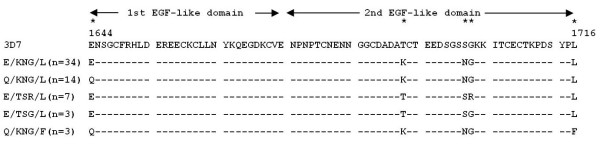
**Haplotypes of the C-terminal fragment of merozoite surface protein-1 (*Pf*MSP-1**_**19**_**) from *Plasmodium falciparum *populations in Thailand.** The arrows indicate the epidermal growth factor (EGF)-like domains 1 and 2 [21]. Asterisks (*) indicate the non-synonymous amino acid substitutions. Numbers above the MSP-1 amino acid sequence of the *P. falciparum* 3D7 indicated the positions after Miller *et al.*[14]. Dashes (-) represent identical amino acid sequences. Number in bracket indicates the number of parasite isolates with that specific *Pf*MSP-1_19_ haplotype.

Nucleotide sequences of *P. falciparum msp-1* gene block 17 (corresponding to nucleotide positions 4990 to 5208 after Tanabe *et al.*[15]) encoding the C-terminal fragment of the MSP-1 protein (MSP-1_19_, amino acid positions 1644 to 1716 after Miller *et al.*[14]) were obtained from 61 parasite isolates from five geographical locations in Thailand between 2002 and 2008, as described in the *Materials and Methods*. Five polymorphic sites (2.28% of the 219 nt. fragments analysed) at nucleotide positions 4990 G/C, 5132 C/A, 5159 G/A, 5161 G/A and 5206 C/T were detected (see Additional file 2 for the sequence alignment). The nucleotide substitution frequency at positions 4990, 5132, 5159, 5161 and 5206 was G/C (43/18;70.5/29.5%), C/A (10/51; 16.4/83.6%), G/A (51/10; 83.6/16.4%), G/A (55/6; 90.2/9.8%) and C/T (58/3; 95/5%), respectively. These nucleotide substitutions results in non-synonmous amino acid substitutions at positions 1644 E (*G*AA)/ Q (*C*AA), 1691 T (A*C*A)/K (A*A*A), 1700 S (A*G*C)/N (A*A*C), 1701 G (*G*GA)/ R (*A*GA) and 1716 L (*C*TT)/ F (*T*TT) (the italicized letter indicated the polymorphic site). The frequency of amino acid substitutions at each site was E1664Q (43/18; 70/30%), T1691K (10/51; 16/84%), S1700N (10/51; 16/84%), R1701G (6/55; 10/90%) and L1716F (58/3; 95/5%). The *Pf*MSP-1_19_ fragment contained nine conserved Cys residues. The first amino acid substitution (E1664Q) was located between the second and the third Cys residues in the first putative EGF-like motif, while the other substitutions occurred in the second EGF-like motif of *Pf*MSP-1_19_ (Figure 1). Therefore, the *Pf*MSP-1_19_ amino acid sequences yielded five distinct haplotypes: E/KNG/L, E/TSR/L, Q/KNG/L, E/TSG/L (3D7 type) and Q/KNG/F, all of which have been reported previously [37-51].

### Prevalence and distribution patterns of MSP-1_19_ in geographically isolated Thai *P. falciparum* populations

**Figure 2:**
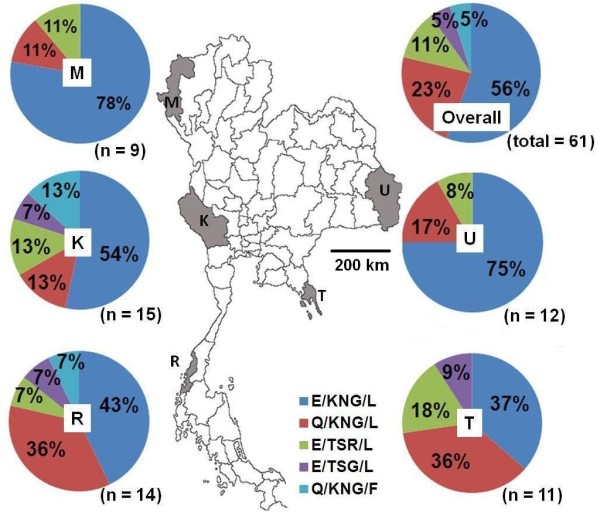
**Allelic diversity of the C-terminal fragment of *Pf*MSP-1**_**19 **_**in *Plasmodium falciparum *populations in Thailand.** Sampling sites were at the borders of Thailand and three neighboring countries: Ubon Ratchathani (U), located at the Laos-Thailand border, Trat (T), located at the Cambodian-Thailand border, and Mae Hong Son (M), Kanchanaburi (K) and Ranong (R), located at the Myanmar-Thailand border. Numbers (n) of the total parasite isolates in Thailand (overall) or the parasites from each locality are displayed in the bracket. Numbers in pie charts represent the percentage of each MSP-1_19_ haplotype (E/KNG/L, dark blue; Q/KNG/L, red; E/TSR/L, green; E/TSG/L, purple; QKNG/F, light blue).

Figure 2 shows the overall prevalence and distribution pattern of *Pf*MSP-1_19_ haplotypes in the five geographical locations of Mae Hong Son (n = 9), Kanchanaburi (n = 15) and Ranong (n = 14), located at the Thailand-Myanmar border, Ubon Ratchathani (n = 12) located at the Thailand-Laos border, and Trat (n = 11), located at the Thailand-Cambodia border (see also Additional file 1). Of the five haplotypes identified, E/KNG/L, Q/KNG/L and E/TSR/L were the most prevalent and were found in all sampled sites, representing 57%, 23% and 11% of the overall haplotypes investigated, respectively.

These results also revealed that there were slight differences in the levels of genetic diversity, based upon the number of *Pf*MSP-1_19_ haplotypes, among the different geographical populations (Figure 2). *P. falciparum* populations in Mae Hong Son and Ubon Ratchathani were the least genetically diverse group of parasites, with only three haplotypes identified. E/KNG/L was the major *Pf*MSP-1_19_ haplotype at both sites, and represented 75–78% of the total haplotypes. In contrast, all the five *Pf*MSP-1_19_ haplotypes were prevalent in the other three *P. falciparum* populations. Only in Trat was four haplotypes (all except for haplotype Q/KNG/F) identified. In these three sites, the E/KNG/L, Q/KNG/L and E/TSR/L haplotypes constituted more than 80% of the total haplotypes investigated. Thus, the level of genetic diversity varied according to the geographical location of the parasite populations, which might reflect variation in the transmission intensity and multiplicity of human malaria infections between sampling sites [66].

To further determine whether the geographically isolated parasite populations are genetically isolated, pair-wise inter-population comparisons were performed for each parasite population using the Wright’s fixation index (F_st_). In this test, the distribution patterns of *Pf*MSP-1_19_ haplotypes in Tak province (collected in 1995), which is located between Kanchanaburi (the region in which the parasite genetic diversity was highest) and Mae Hong Son (the region in which the parasite genetic diversity was lowest), from two previous studies were also included. One consisted of 8 E/TSR/L, 25 E/KNG/L, 12 Q/KNG/L and 3 Q/KNG/F haplotypes (total = 48) [39], while the other comprised 14 E/TSR/L, 39 E/KNG/L, 13 Q/KNG/L, 4 Q/KNG/F and 2 E/TSG/L haplotypes (total = 72) [38]. As shown in Table 1, the F_st_ values from the pairs of all the six parasite populations were low and non-significant (*p* > 0.05). Accordingly, the *P. falciparum* parasite populations (or at least the *Pf*MSP-1_19_) circulating in this region are genetically homogeneous, suggesting gene flow between allopatric populations of the malaria parasites near the borders of Thailand with Myanmar, Laos and Cambodia.

**Table 1:** **Pairwise F**_**st **_**values of *Pf*** **MSP-1**_**19 **_**haplotypes between geographically isolated *Plasmodium falciparum *** **populations in Thailand**

	**Mae Hong Son**	**Ubon Ratchathani**	**Kanchanaburi**	**Trat**	**Ranong**
Ubon Ratchathani	-0.09985	-			
	(*p* = 0.99)				
Kanchanaburi	-0.04761	-0.02984	-		
	(*p* = 0.77)	(*p* = 0.56)			
Trat	-0.01113	0.01017	-0.05754	-	
	(*p* = 0.41)	(*p* = 0.23)	(*p* = 0.73)		
Ranong	0.00241	-0.00344	-0.04347	-0.04642	-
	(*p* = 0.42)	(*p* = 0.39)	(*p* = 0.72)	(*p* = 0.74)	
Tak*	-0.02637	-0.01682	-0.03795	-0.03720	-0.02997
	(*p* = 0.51)	(*p* = 0.55)	(*p* = 0.89)	(*p* = 0.67)	(*p* = 0.71)
Tak**	-0.02075	-0.00632	-0.02612	-0.01521	-0.0010
	(*p* = 0.4	(*p* = 0.46)	(*p* = 0.89)	(*p* = 0.66)	(*p* = 0.71)

Frequency of the *Pf*MSP-1_19_ haplotypes in Tak were derived from two studies by (*) Sakihama *et al.*, [38] and (**) Sakihama *et al.*, [39]. All F_st_ values were non-significant (*p* > 0.05).

### Conserved patterns of MSP-1_19_ haplotypes in three transmission seasons

**Figure 3:**
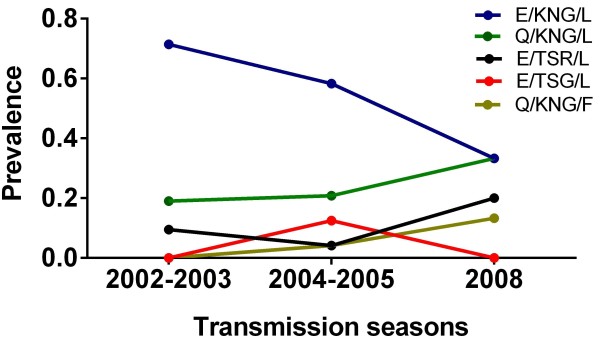
**Prevalence of the C-terminal fragments of *Pf*MSP-1**_**19 **_**in Thai *Plasmodium falciparum *populations over time.***P. falciparum* samples were collected in three transmission seasons: Jun 2002–Oct 2003, Jun 2004–Dec 2005 (but including one sample collected in 2006, see Materials and Methods) and Jan 2008–Dec 20008. MSP-1_19_ haplotypes: E/KNG/L, blue; Q/KNG/L, green; E/TSR/L, black; E/TSG/L, red; Q/KNG/F, yellow.

The prevalence of MSP-1_**19**_ haplotypes in three transmission seasons is shown in Figure 3. Of the 61 *msp*-1 sequences analysed, 21 sequences were obtained in a 15-month period between Jun 2002–Oct 2003 and contained three haplotypes (E/KNG/L, E/TSR/L and Q/KNG/L); 25 sequences were obtained in an 18-month period between Jun 2004–Dec 2005 (plus one sample collected in early 2006) and contained all five haplotypes; and 15 sequences were collected in a 12-month period between Jan–Dec 2008. As seen in Figure 3, the E/KNG/L and Q/KNG/L haplotypes were the two most prevalent haplotypes in all three seasons, with cumulative frequencies of 60–80% of the haplotypes identified throughout the study years. However, over this time period the E/KNG/L haplotype decreased in frequency whilst the Q/KNG/L haplotype increased, with the relative E/KNG/L: Q/KNG/L proportion changing from 3.5:1 in Jun 2002–Oct 2003 to 1:1 in Jan 2008–Dec 2008, indicating longitudinal variation in the frequencies of the prevalent haplotypes. In contrast, the frequencies of each of the three minor haplotypes remained low (>10%) and relatively stable throughout the three-year period.

Pair-wise inter-population comparison of the *Pf*MSP-1_19_ haplotypes between years was compared using the Wright’s fixation index (F_st_), and this revealed low and non-significant F_st_ values (*p* > 0.05) (Table 2), suggesting a similar MSP-1_19_ haplotype distribution between the parasite populations in successive years. Furthermore, when the haplotypes of MSP-1_19_ from Tak province (collected in 1995 [38,39]) was included in the analysis, the results also revealed a similar haplotype distribution in 1995 and the other study periods (see Table 2). Thus, the frequencies of the individual haplotypes were potentially stable during the four transmission seasons.

**Table 2:** **Pairwise F**_**st **_**values of *Pf*** **MSP-1**_**19 **_**haplotypes in *Plasmodium falciparum *** **populations in the sampling years 1995, 2002-2003, 2004-2005 and 2008**

	Population in 1995*	Population in 1995**	**Population in Jun 02-Oct 03**	**Population in Jun 04-Dec 05**
Population in Jun 02-Oct 03	0.00575	-0.00694	*	
	(*p* = 0.30)	(*p* = 0.40)		
Population in Jun 04-Dec 05	-0.00268	-0.01639	-0.02722	*
	(*p* = 0.92)	(*p* = 0.71)	(*p* = 0.73)	
Population in Jan 08-Dec 08	0.02685	-0.02287	0.04219	0.00518
	(*p* = 0.16)	(*p* = 0.59)	(*p* = 0.14)	(*p* = 0.30)

Frequency of the *Pf*MSP-1_19_ haplotypes in 1995 were from two studies by (*) Sakihama *et al.*, [38] and (**) Sakihama *et al.*, [39]. All F_st_ values were non-significant (*p* > 0.05).

### Selection signature on *msp-1* gene block 17 locus

**Figure 4:**
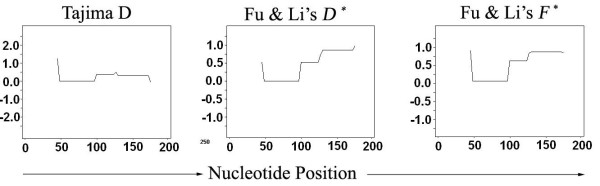
Sliding window plots of Tajima’s *D*, Fu & Li’s D* and Fu & Li’s F* for the *Pfmsp-1 *gene block 17. Window length is 90 bp and step size is 3 bp. No evidence of a significant departure from neutrality or of diversifying selection was observed.

The geographically separate and so potentially isolated populations of *P. falciparum* in Thailand were found to not be significantly heterogeneous at the *Pf*MSP-1_19_ locus, suggesting that the different *Pf*MSP-1_19_ haplotypes may be maintained by balancing selection. Therefore, intra-population based analyses (Tajima’s D test, Fu & Li’s D^*^ test and Fu & Li’s F^*^ test) were used to evaluate the selection signature on the *Pf*MSP-1_19_ locus. No significant positive D value (Tajima’D = 0.4082; Fu & Li’s D^*^ = 1.079 and Fu & Li’s F^*^ = 1.016) was found for the entire block 17 coding region. In addition, although a sliding window analyses of Tajima’s D test, Fu & Li’s D^*^ test and Fu & Li’s F^*^ test revealed positive D values for the EGF-1 and EGF-2 domains, they were not statistically significant (Figure 4). A comparison of the ratio of synonymous to non-synonymous substitutions (K_a_/K_s_) was not analysed because the number of synonymous differences (K_s_) was too low (zero). Overall, the polymorphisms at the C-terminal fragment of the *msp-1* gene block 17 were less likely to be under strong immune selection, suggesting a functional constraint that limits the allelic diversity of the sequences. Taken together, the polymorphisms detected in *Pf*MSP-1_19_ were neutral and highly conserved in the geographically isolated, but genetically homogenous *P. falciparum* populations in Thailand.

## Discussion

The 19-kDa antigenic domain of *Pf*MSP-1 is considered the major target component for the developmetn of human blood stage malaria vaccines [13]. Therefore, the analysis of the sequence diversity of the *msp*-*1* gene block 17 that encodes *Pf*MSP-1_19_ has gained considerable attention in the global malaria research community, leading to the identification of its polymorphism level and haplotypes [37-51]. *Pf*MSP-1_19_ sequences have been primarily classified into two prototypic allelic groups: (1) the MAD20 prototypic allelic group (E/TSR/L haplotype) and (2) the Wellcome prototype allelic group (Q/KNG/L haplotype) [15]. In addition, other variant haplotypes (E/KNG/L, E/TSG/L and Q/KNG/F) have also been reported in natural populations of *P. falciparum* worldwide [37-51]. These haplotypes were proposed to be generated through recombination events between the two prototype alleles and due to mutations [42]. The analysis of *Pf*MSP-1_19_ sequence diversity in Thailand was previously conducted in a single locality in Tak, located on the western border of Thailand and identified at least five distinct haplotypes (E/KNG/L, E/TSR/L, Q/KNG/L, E/TSG/L and Q/KNG/F) [38]. However, it is not known whether the extent of *Pf*MSP-1_19_ sequence variation is common in other populations of *P. falciparum* in Thailand or whether the distribution pattern of these haplotypes is conserved. This question formed the basis of the present study, which aimed to screen for polymorphism and evaluate the haplotypes of the *msp-1* gene block 17 from different geographical regions in Thailand.

Nucleotide sequence analysis of the *msp*-1 gene block 17 was performed on 61 Thai *P. falciparum* isolates originating from five endemic areas; Mea Hong Son, Kanchanaburi and Ranong, along the borders of Thailand and Myanmar, Trat at the border of Thailand and Cambodia, and Ubon Ratchathani, at the border of Thailand and Laos. These areas were among the top 15 provinces with the highest numbers of *P. falciparum* malaria cases in Thailand [67]. The five conserved polymorphic SNP sites that caused non-synonymous amino acid substitutions in the *Pf*MSP-1_19_ sequences (haplotypes E/KNG/L, E/TSR/L, Q/KNG/L, E/TSG/L and Q/KNG/F) previously identified in Tak [38,39,51] and in other endemic regions worldwide [37,40-50], were found with no new haplotypes. Consistent with previous observations, these results reported here indicated that the allelic diversity of the *msp*-1 gene block 17 in Thai *P. falciparum* populations was limited.

The levels of genetic diversity of the *msp-1* sequences in Thai populations varied according to the geographical locations. The lowest genetic diversity was detected in *P. falciparum* populations in Mae Hong Son and Ubon Ratchathani, with only three *Pf*MSP-1_19_ haplotypes of being found, compared to four and five in Kanchanaburi and Ranong, respectively. Variation in the levels of genetic diversity in geographically isolated populations may be due to variation in the multiplicity of infection, malaria incidence, transmission intensity and levels of gene flow between the parasite populations [59,66]. Malaria incidence rates showed a high spatial heterogeneity across the geographical regions in Thailand, with the highest rates in the regions near the borders of Thailand with Myanmar and Cambodia and lower rates in the regions near the Thailand-Laos border [68]. In addition, multiple or polyclonal infections with *P. falciparum* were highly prevalent at the Thailand-Myanmar border region in Kanchanaburi compared to at the Thailand-Laos borders [59]. Elevated levels of mixed genotype infections may well increase the frequency of recombination events of the *msp-1* alleles in the mosquito and subsequently a high diversity of *msp-1* would be maintained in Thai populations [66].

When the diversity and distribution patterns of MSP-1_19_ haplotypes in Thai populations in the present study were compared with results from a previous study based upon microsatellite typing [59], no genetic differentiations was found between sub-populations in Mae Hong Son and Ubon Ratchathani, Mae Hong Son and Trat, Mae Hong Son and Ranong, Kanchanaburi and Ranong, and Ubon Ratchathani and Trat. However, the microsatellite analysis revealed a significant level of differentiation between sub-populations in Kanchanaburi and three other provinces (Mae Hong Son, Ubon Ratchathani and Trat), and in Ranong and two other provinces (Ubon Ratchathani and Trat). The discrepancy could be partly attributed to the differences in the sample size and the types of genetic markers used. Whether genetic differentiations exist in Thai sub-populations will require further investigations using a larger size of samples and additional markers, and this is an area of our current research.

The level of genetic differentiation of *Pf*MSP-1_19_ in populations in Thailand, as detected using F-statistics (F_st_), revealed no significant differences between the *P. falciparum* populations located at the Thai borders with Myanmar, Laos and Cambodia. Accordingly, there may be no barriers that limit gene flow between geographically isolated *P. falciparum* populations in Thailand. This would likely reflect that there are numerous host (human) movements within and into Thailand from neighboring countries from immigrants who live across the borders [69,70]. For example, it was reported that in 2006 there were 30,338 malaria cases found in native Thais compared to 36,313 malaria cases from foreigners living in Thailand [71]. A cross-sectional survey showed that up to 75% of the malaria incidences in regions along the Thai borders were imported cases [67]. The majority of malaria-infected migrant workers were from Cambodia, Myanmar and Laos. Short-term and permanent migrations of infected humans, such as immigrant workers, may play a key role in the development and spread of anti-malaria drug resistance. This study also indicated that the Thai parasite populations along the three national borders are not genetically separated (and so may be mixed) and so are potentially subject to gene flow of novel phenotypes, most probably from Myanmar and Cambodia, where malaria transmission rates are high and multiclonal infections are prevalent. Gene flow within parasite populations may play an important role in shaping their population structure. Indeed, previous studies showed that the emergence of artemisinin resistance in *P. falciparum* occurred around the Thai-Cambodia border in 2009 and now has recently been found at the Thai-Myanmar border in 2011. The results from this study suggest that it is likely that the artemisinin resistant strains of *P. falciparum* could also spread to other regions of Thailand.

The longitudinal survey of MSP-1_19_ haplotypes in four transmission seasons (from 1995 to 2008) revealed that the overall frequency of individual *Pf*MSP-1_19_ haplotypes was relatively stable, although the two major haplotypes (E/KNG/L and Q/KNG/L) inversely fluctuated over time. This result was also seen in a previous longitudinal survey in Mali [44]. Comparison of the frequency of these *Pf*MSP-1_19_ haplotypes in endemic regions worldwide indicated a dramatic distribution pattern and frequency of *Pf*MSP-1_19_ haplotypes. The high prevalence of the E/KNG/L and Q/KNG/L haplotypes reported in Thailand has also been reported in the Yunan and Hainan provinces in China and Vietnam [37,46], suggesting that *P. falciparum* populations in the Greater Mekong Sub-region (GMS) (China, Thailand, Laos, Myanmar, Cambodia and Vietnam) could be genetically related. Whether the parasite populations in GMS are genetically homogeneous will need to be evaluated by large-scale genotyping approaches using genome-wide genetic markers (e.g. microsatellites and SNPs, see [72]). Similar distribution patterns of *Pf*MSP-1_19_ in samples from the GMS have also been detected in African countries, such as Kenya, Tanzania and Mali [42-44]. In contrast, the three rare *Pf*MSP-1_19_ haplotypes (E/TSR/L, ETSG/L and Q/KNG/L) appeared to be the dominant haplotypes in other regions, such as South Asia (India and Iran), South America (Brazil and Peru), Vanatu and the Solomon Islands [39-41,50]. By providing information about the prevalence and spatiotemporal dynamics of *Pf*MSP-1_19_ haplotypes, this study could help inform choices about which *Pf*MSP-1_19_ haplotypes to formulate future vaccines from and to allow a more accurate interpretation of the efficacy of current formulations of *Pf*MSP-1_19_ based vaccines being tested in clinical trials. Currently, MSP-1 sequences from *P. falciparum* K1 (Q/KNG/L) are being formulated as components of the multivalent blood stage vaccine *Pf*CP-2.9 [35]. If the immunity conferred by such vaccines is allele specific, the *Pf*CP-2.9 vaccine would be advantageous for populations in the GMS and in Africa, but not in South America and the Asian Pacific Islands.

It is possible that the limited allelic diversity in the MSP-1_19_ sequences may be due to positive selection or balancing selection, as has been demonstrated for other regions of MSP-1 [22]. However, the Tajima’s D test, Fu & Li’s D^*^ test and Fu & Li’s F^*^ test all failed to reveal any statistically significant evidence for strong positive or balancing selection of the C-terminal fragments of *Pf*MSP-1_19_ in these Thai samples. The most probable explanation for the limited diversity in *Pf*MSP-1_19_ is the functional constraint of the protein. MSP-1 is considered to play an important role in erythrocyte entry by the merozoite [13]. Secondary processing of *Pf*MSP-1 to *Pf*MSP-1_19_ is a prerequisite for merozoite invasion [73]. The *Pf*MSP-1_19_ fragment possesses different epitopes and variation in the motifs alters the conformational structure markedly [74,75]. It may be that each motif has a unique association with other parts of MSP-1 and these differential intermolecular associations have implications in evading the host immune responses by masking the target epitopes in the 19-kDa domain [76]. Thus, selective constraints in the light of the host immune responses and functional constraints at the protein level may be co-operative to maintain the limited diversity of the MSP-1_19_ amino acid sequences.

## Conclusion

This study extends the understanding and current knowledge of the variation and prevalence of *Pf*MSP-1_19_ polymorphisms in natural populations of *P. falciparum* in Thailand. The data supports the view that *P. falciparum* populations in the GMS region are genetically diverse. Analysis of the spatiotemporal patterns of MSP-1 haplotypes indicated that E/KNG/L and Q/KNG/L were the major haplotypes in Thailand, which were also highly prevalent in populations from other Southeast Asian countries, China and Africa. This study provides the basis for the accurate measurement and interpretation of the population structure and dynamics of malaria parasites that is critical for monitoring the population responses to MSP-1 based vaccines in clinical trials.

## Competing interests

The authors declare that they have no competing interests.

## Authors’ contributions

PS carried out the molecular genetic work, interpretation of data and drafted the manuscript. SP performed data analysis and wrote the manuscript. NS and AS were responsible for parasite cultivation. MK and RB carried out the statistical analysis and guidance for data interpretation. PH provided conceptual framework for the project, guidance for the interpretation of data, participated in the manuscript preparation, revision and coordination. All authors read and approved the final manuscript.

## Supplementary Material

Additional file 1 **The origin and date of collection of the human malaria parasite *Plasmodium falciparum *** **used in the present study.** Asterisks (*) indicate samples collected between 2002 and 2006 that were previously genotyped by 12 microsatellite loci and confirmed to be independent clones [59]. Click here for file

Additional file 2 **Nucleotide sequence alignment of the *Plasmodium falciparum *** **merozoite surface protein-1 *(msp-1) *** **gene block 17.** Data shows the representative five variants (haplotypes E/KNG/L, Q/KNG/L, E/TSR/L, E/TSG/L and Q/KNG/L) of the 61 samples analysed. The nucleotide positions are presented on top of the *P. falciparum* 3D7 sequence (NCBI accession number: XM_001352134; [14]). Nucleotides in green are potential N-glycosylation recognition sites [21]. Arrow indicates the first and second epidermal growth factor like domains [38]. Asterisks (*) show the five conserved polymorphic nucleotides (red), located at positions 4990, 5132, 5159, 5161 and 5206, respectively. Dashed lines indicate nucleotides that are identical to those of 3D7. Click here for file
