# Supplementary material for: Diversity and population structure of Plasmodium falciparum in Thailand based on the spatial and temporal haplotype patterns of the C-terminal 19-kDa domain of merozoite surface protein-1
Source: Malar J. 2014 Feb 12;13:54. doi: 10.1186/1475-2875-13-54 (PMC3931489; doi:10.1186/1475-2875-13-54)
Supplement: Additional file 2 — Nucleotide sequence alignment of the Plasmodium falciparum merozoite surface protein-1 (msp-1) gene block 17. Data shows the representative five variants (haplotypes E/KNG/L, Q/KNG/L, E/TSR/L, E/TSG/L and Q/KNG/L) of the 61 samples analysed. The nucleotide positions are presented on top of the P. falciparum 3D7 sequence (NCBI accession number: XM_001352134; [14]). Nucleotides in green are potential N-glycosylation recognition sites [21]. Arrow indicates the first and second epidermal growth factor like domains [38]. Asterisks (*) show the five conserved polymorphic nucleotides (red), located at positions 4990, 5132, 5159, 5161 and 5206, respectively. Dashed lines indicate nucleotides that are identical to those of 3D7. [file 1475-2875-13-54-S2.pdf]

Block 16/ block 17

```
                                                                4812                                             *
3D7          ATGTTAAAC ATTTCACAAC ACCAATGCGT AAAAAAACAA TGTCCAGAAA ATTCTGGATG
E/KNG/L                                                       G--- ----------
Q/KNG/L                                                       C--- ----------
E/TSR/L                                                       G--- ----------
E/TSG/L                                                       G--- ----------
Q/KNG/F                                                       C--- ----------
```

1st **EGF-like domain**

```
             4872
3D7          TTTCAGACAT TTAGATGAAA GAGAAGAATG TAAATGTTTA TTAAATTACA AACAAGAAGG
E/KNG/L      ---------- ---------- ---------- ---------- ---------- ----------
Q/KNG/L      ---------- ---------- ---------- ---------- ---------- ----------
E/TSR/L      ---------- ---------- ---------- ---------- ---------- ----------
E/TSG/L      ---------- ---------- ---------- ---------- ---------- ----------
Q/KNG/F      ---------- ---------- ---------- ---------- ---------- ----------
```

```
             4932
3D7          TGATAAATGT GTTGAAAATC CAAATCCTAC TTGTAACGAA AATAATGGTG GATGTGATGC
E/KNG/L      ---------- ---------- ---------- ---------- ---------- ----------
Q/KNG/L      ---------- ---------- ---------- ---------- ---------- ----------
E/TSR/L      ---------- ---------- ---------- ---------- ---------- ----------
E/TSG/L      ---------- ---------- ---------- ---------- ---------- ----------
Q/KNG/F      ---------- ---------- ---------- ---------- ---------- ----------
```

2nd **EGF-like domain**

```
             4992    *                               * *
3D7          AGATGCCACA TGTACCGAAG AAGATTCAGG TAGCAGCAGA AAGAAAATCA CATGTGAATG
E/KNG/L      --------A- ---------- ---------- -----A-G-- ---------- ----------
Q/KNG/L      --------A- ---------- ---------- -----A-G-- ---------- ----------
E/TSR/L      --------C- ---------- ---------- -----G-A-- ---------- ----------
E/TSG/L      --------C- ---------- ---------- -----G-G-- ---------- ----------
Q/KNG/F      --------A- ---------- ---------- -----A-G-- ---------- ----------
```

```
             5052                     *
3D7          TACTAAACCT GATTCTTATC CACTTTTCGA TGGTATTTTC TGCAGTTCCT
E/KNG/L      ---------- ---------- --C--
Q/KNG/L      ---------- ---------- --C--
E/TSR/L      ---------- ---------- --C--
E/TSG/L      ---------- ---------- --C--
Q/KNG/F      ---------- ---------- --T--
```
